# Validation of a core patient-reported-outcome measure set for operationalizing success in multimodal pain therapy: useful for depicting long-term success?

**DOI:** 10.1186/s12913-018-2911-6

**Published:** 2018-02-17

**Authors:** Carolin Donath, Christa Geiß, Christoph Schön

**Affiliations:** 1 Center for Health Services Research in Medicine, Department of Psychiatry and Psychotherapy, Friedrich-Alexander-Universität Erlangen-Nürnberg, Schwabachanlage 6, 91054 Erlangen, Germany; 2 Interdisciplinary Pain Center, University Clinic Erlangen, Friedrich-Alexander-Universität Erlangen-Nürnberg, Krankenhausstr. 12, 91054 Erlangen, Germany

**Keywords:** (MeSH): Quality indicators, Health care, Patient-reported-outcome measures, Patient outcome assessment, Therapeutics, Pain management, Validation studies, Quality assurance, Health care

## Abstract

**Background:**

The study aims to validate a previously developed and published combined success criterion for patients after multimodal pain therapy (Donath et al., BMC Health Serv Res 15:272, 2015). The criterion classifies treated patients as successful in the long term on the basis of pain severity, disability through pain, depressiveness, and health-related quality of life.

**Methods:**

Routine longitudinal data of 135 pain patients treated with multimodal pain therapy in 2014–2015 at the Interdisciplinary Pain Center of the University Clinic Erlangen were available at baseline, therapy start, therapy end, and 12 months after treatment. Patients were, on average, 51.0 (SD 11.1) years old and to 63.7% female, two thirds were employed (66.7%). We conducted an analysis of concurrent validity (with: pain severity, disability through pain, depressiveness, mental and physical quality of life), criterion validity (with disability days, self-rated success), convergent validity (with stress, anxiety, well-being), and discriminant validity (with chronicity of pain, comorbidity), objectivity, and reliability. Statistically, descriptive and inference statistics, graphical methods and MANOVAs were used.

**Results:**

Patients classified as successful had significantly better values on the 5 variables demonstrating concurrent validity (all *p* < .001), significantly fewer Disability days (M = 15.31 (SD = 23.15) vs. M = 26.75 (SD = 29.15)); t (133) = 2.308; *p* = .024, less Anxiety (Pillai-Spur: F (3, 131) = 2.972, *p* = .034), less Stress (Pillai-Spur: F (3, 131) = 9.907, *p* < .001), and better Well-being (Pillai-Spur: F (3, 131) = 9.594, *p* < .001) 12 months after treatment than patients classified as not successful. The Spearman correlation between success classification and Chronicity stage was .094 (*p* = .280).

**Conclusion:**

We demonstrated the validity of the combined success criterion with long-term data in addition to confirming the reliability and objectivity of the criterion. Future research might consider identifying predictors of success in multi-modal pain therapy.

**Electronic supplementary material:**

The online version of this article (10.1186/s12913-018-2911-6) contains supplementary material, which is available to authorized users.

## Background

Multimodal pain therapy can be counted as an effective interdisciplinary complex intervention. However, the comparability of results of such therapies has been impaired by a widely heterogenic use of outcome instruments [1]. In fact, a systematic review showed that no outcome domains were measured consistently across all of the 70 studies in the review [1, 2]. There are efforts to define a core set of patient-relevant outcome variables for multimodal pain therapy, which is an intensive, time-consuming process that requires the consensus of different expert groups [3] and takes places in ongoing research projects. The Core Outcome Measures in Effectiveness Trials (COMET) group uses the Delphi method [4] to develop core outcome sets for clinical trials – not pain-specific but for different patient populations.

In the meantime, we used the existing and, to date, consented “German Pain Questionnaire” [5] to define a core outcome set for multimodal pain therapy. This core set corresponds in content with the most widely used outcome domains identified by Deckert and colleagues [1]. The combined outcome measurement consists of five single domains, all operationalized via patient-reported outcomes included in the German Pain Questionnaire. The domains are: pain severity, pain disability, depressiveness, and health-related quality of life (physical and mental). It consequently allows a dichotomous decision to be made about whether a patient has successfully completed multimodal pain therapy or not [6]. A first validation was conducted and published.

However, the tool for operationalizing success has yet to be validated with long-term data, a shortcoming that has been discussed. Thus, the present study is aimed at validating the previous pilot version of the combined success criterion with long-term (12-month follow-up) data. Furthermore, this validation study also presents additional analyses regarding convergent, discriminant, concurrent, and criterion validity as well as reliability and objectivity. The pilot version published in 2015 reported data on predictive validity and convergent validity with other constructs (FESV-scales) [6].

### Aims

The aim of the study was to validate the core patient-reported-outcome measurement set (also called the combined success criterion) for classifying pain patients who received multimodal pain therapy with respect to their long-term success. For validation purposes concurrent validity, criterion validity, convergent and discriminant validity was assessed with objectivity and reliability also being evaluated.

## Methods

### Design

The data for the study are routine, longitudinal data from patients at the interdisciplinary pain center of the University Clinic in Erlangen. The patients participated in a semi-inpatient five-week multimodal pain therapy and were assessed by trained pain therapists and with self-report questionnaires at four time points: before the patients began therapy (= at screening), at the start and end of treatment, and 12 months later (= 12-month follow-up). The average time passed between screening and start of therapy was 3 months. To analyze the validity of the combined success criterion, we used the 12-month follow-up data.

The data analyzed here were collected within the time frame of January 1, 2014 to June 30, 2015. The term routine data indicates that no extra data were collected for the study. Patients were asked to fill out a paper-pencil questionnaire which took approximately 60 min to complete. At the time points before the therapy and at follow-up, the collection took place at home, while at beginning and end of therapy patients assessments took place during the consultations. Routine data were used in the initial development and publishing of the success criterion [6] (first data set) and also for the current validation (second data set). Thus, this validation analysis was undertaken with different patients than the development and pilot testing. The patients were informed about the treatment and the kind of data collection that would accompany their therapy. They provided written consent to be treated and to have their data collected and anonymously analyzed for scientific use. The data were anonymized, and the person who implemented the data analysis did not see any person-related information in the data and also did not see any patients in person so that it would not be possible to form a connection between sensitive data and real persons. The data were treated according to the German and Bavarian legislative rules for data protection. Routine data collection accompanies therapeutic action at the University Clinic Erlangen as a matter of quality assurance. This is in accordance with the ethical commission of the Friedrich-Alexander-Universität Erlangen-Nürnberg.

### Sample

A sample of *N* = 135 was available for analysis. Data from patients with a chronic pain condition followed-up 12 months after enrollment in the 5-week multimodal pain therapy were used to validate the combined success criterion, which was based on patient-reported outcomes. The sample consisted of 63.7% women; the mean age was 51.0 (SD 11.1) years. 85.9% of the patients were German, 5% had a migration background, 62.2% were married, and 80.0% of the whole sample did not live alone. The majority (40.0%) had a general secondary school certificate (9 years) = “Hauptschulabschluss,” and one seventh of the patients had a University diploma (14.1%). The largest part of the sample had completed an apprenticeship (88.3%), and two thirds of the sample were employed (66.7%). However, 36.7% were on sick leave when the data were collected, and more than one fourth of the whole sample was permanently classified as sick and thus not working. A total of 15.6% of the sample received retirement payments. Almost half of the sample was officially recognized as disabled (42.7%), and 10.1% had filed a claim to receive a confirmation for a higher grade of disability. The majority of the patients reported suffering from pain for more than 5 years (39.3%), and an additional one fourth of the sample reported that they had been in pain for 2 to 5 years (24.4%). The mean number of pain days in the 3 months before treatment was 81.9 (SD 21.9), the mean number of physician assessments because of pain was 2.7 (SD 4.0), and the mean number of physiotherapist assessments because of pain was 3.0 (SD 6.0) in the 3 months before multimodal pain therapy. In Additional file 1 the baseline characteristics of the sample are depicted group-specific for long-term responders and non-responders.

### Instruments

All instruments used in this study were part of the German Pain Questionnaire, a validated collection of scales and items that is used in all pain therapy centers in Germany as part of the core data set assessed for internal and external quality assurance. The instruments we included were negotiated by the “Deutsche Schmerzgesellschaft” section of the “International Association for the Study of Pain” and have been examined in validation studies ([5]: pages 6&7, [7]). We used the revised version from 2012 here.

#### Pain severity

Subjective pain intensity is assessed with a visualized numeric rating scale with the endpoints “no pain” and “most intensive pain imaginable.” The mean of the three pain intensity ratings (actual, average, maximum) was taken and multiplied by 10, yielding a value between 0 and 100 ([5]: page 13, [8]). The pain intensity assessment is part of the internationally used Korff-Graduation for grading the global severity of a chronic pain condition [9].

#### Disability through pain

Also part of the Korff-Graduation for rating the global severity of pain [9] and derived from the Multidimensional Pain Inventory [10], patients rate how much the pain has interfered with their ability to perform activities of daily living, work, and spare-time/social activities. Each item is graded by the patient on a numeric rating scale ranging from 0 to 10, and a mean value is computed ([5]: page 14).

#### Disability days

We assessed the self-rated number of days that patients felt unable to take part in their usual activities during the last 3 months at the follow-up assessment. This item is also part of the global severity rating according to Korff [9].

The three named constructs are part of the Chronic Pain Grade Questionnaire (CPGQ), which is reported to have satisfactory internal consistency values and also an acceptable retest reliability [11].

#### Depressiveness

We assessed depressiveness with a self-report scale that is part of the DASS (Depression, Anxiety, and Stress Scale [12, 13]) consisting of seven items with a very acceptable internal consistency of >.90 (Cronbach’s alpha). The DASS is counted as a sensitive screening instrument for depression in samples of pain patients [5].

#### Anxiety

We assessed anxiety with a self-report scale that is also part of the DASS [12, 13] consisting of seven items with a satisfactory internal consistency of .80 (Cronbach’s alpha) (according to Nigels & Essau [13]: 0.78–0.82).

#### Stress

We used the third subscale of the DASS [12, 13] to assess the construct of individual burden through stress. This subscale has an internal consistency of .87 ([13]: 0.81–0.89).

#### Well-being

We assessed the subjective habitual well-being of the pain patients with the FW-7 (The Marburg Questionnaire on Habitual Well-being) [14, 15], a seven-item scale on which the patients rated their general well-being in the last 14 days. The internal consistency has been reported to be between 0.87 und 0.92 (Cronbach’s alpha) [16]. The construct has been reported to be only moderately correlated with Quality of Life and the DASS variables [5]. Thus, the authors concluded that it offers additional information [5].

#### Quality of life

We assessed patients’ physical- and mental-health-related quality of life with a short form of the SF-36 called the SF-12 [17]. The SF-12 is an internationally widely used and (also for pain patients) validated instrument for measuring health-related quality of life that is economical and meaningful (e.g. [18, 19]). The psychometrics are comparable to the long version [18, 20].

#### Self-rated therapy success

We assessed self-rated therapy success at follow-up with a single item: “If you look at it altogether, how do you judge the success of your treatment with us up to now?” The patient chooses one out of five Likert-scaled answer categories.

#### Number of psychic co-morbidities

We summed the number of co-morbidities that were diagnosed by trained pain therapists (medical doctors) and coded as ICD-10 diagnoses. Only psychic co-morbidities were included. The main diagnosis (not co-morbid) was always the predominant chronic pain condition.

#### Chronicity of pain

We assessed chronicity of pain with the Mainz Pain Staging Scale (MPSS) from Gerbershagen [21, 22]. This scale provides two values: the chronicity stage (from 1 to 3) and a sum score that operationalizes the chronicity of the pain condition and its consequences. The content covers aspects of time and place of pain, medication intake behavior and patient career. Each item of the four aspects is evaluated with a number between 1 and 3, a sum score is built and depending on this a chronicity stage is assigned.

### Statistical analysis

The analysis computed for the validation was based on a data set of 135 cases analyzed with SPSS 21. We imputed individual missing data values for *N* = 22 patients using the EM-algorithm [23–26]. Those patients (16.3%) did not send back the follow-up assessment data in spite of 3-times-reminding. For the description of the sample and the descriptive assessment of the combined success criterion at the 12-month follow-up, we computed descriptive statistics (means, standard deviations (SD), and frequencies).

#### Computation of the final combined success criterion (dichotomous variable)

In order to classify the sample according to the dichotomous success criterion (responder/non-responder after 12 months), we computed change values (mean and SD) between the 12-month follow-up and the start of therapy. Each individual was assessed for whether he or she improved by ½ SD or more for each of the five single criteria that were incorporated into the combined success criterion (pain severity, disability through pain, depressiveness, physical- and mental-health-related quality of life). After that, for each individual, we computed the number of single criteria for which the improvement was clinically relevant (at least ½ SD). All patients who showed improvement on four or all five of the single criteria that were used to create the combined success criterion were counted as “responders” or as “successful” and were coded as 1 on the final success criterion variable. All others were classified as “non-responders”/“not successful” and coded as 0 [6]. The reasons for choosing the criterion of ½ SD-change as clinically relevant are published elsewhere including the discussion of its limitations [6]. The numbers of patients fulfilling the different numbers of subcriteria are presented in the results.

#### Statistical methods used for the validation analysis

Table 1 provides an overview of the kinds of validity checks we carried out, the scales or variables we used to evaluate this type of validity in the current study and the statistical procedures applied.

**Table 1:** Overview of variables/scales and types of validity concerning the combined success criterion for multimodal pain therapy

Type of validity	Scales or variables used	Statistical procedure
Concurrent validity	Pain severity Disability through pain Depressiveness Physical-health-related quality of life Mental-health-related quality of life	Descriptive and Inference statistics (t-test) Graph (Overview)
Criterion validity	Self-rated therapy success Disability days	Descriptive and Inference statistics (t-test) Graph (Overview)
Convergent validity	Anxiety Stress Well-being	MANOVAs Graphs (Specific and Overview)
Discriminant validity	Chronicity of pain Number of psychic co-morbidities	Correlations Graphs (Overview)

## Results

### Success criterion multimodal pain therapy – 12-month follow-up data

The previously published combined success criterion incorporating the five domains of pain severity, pain disability, depressiveness, and health-related quality of life (physical and mental) defines success if at least 4 out of the 5 single criteria have improved at least ½ standard deviation [6]. Here, the percentages of “successfully treated” patients 12 months after therapy are presented in Table 2.

**Table 2:** Frequency of fulfilled success criteria (12-month follow-up)

Number of single criteria showing improvement (change of at least ½ SD)		
	Frequency (N)	Percent
0 1 2 3 **4** **5** Total	19 27 35 21 17 16 135	14.1 20.0 25.9 15.6 **12.6** **11.9** 100.0

Bold: Definition of success

**Fig. 1:**
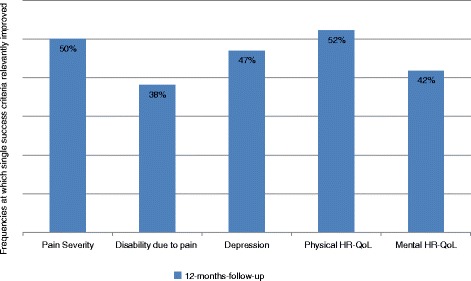
Frequency of success in single domains (at least ½ standard deviation improvement): 12-month follow-up data. HR-QoL: Health-Related Quality of Life

The mean changes (therapy start to follow-up) for all 135 patients were − 9.4 (SD 18.49) for pain severity, − 1.04 (SD 1.98) for disability through pain, − 2.10 (SD 5.07) for depressiveness, + 5.40 (SD 8.18) for physical-health-related quality of life, and + 2.71 (SD 10.34) for mental-health-related quality of life from the start of therapy to the 12-month follow-up. The success rate 12 months after therapy using the criterion was 24.5%. The two most successful single criteria were change in pain severity and increase in physical-health-related quality of life – about half of the treated patients showed a clinically relevant improvement on these two variables 12 months after they completed multimodal pain therapy (Fig. 1).

### Validation of the combined patient-reported-outcome (“PRO”) criterion for success in multimodal pain therapy with 12-month follow-up data

#### Concurrent validity

Patients classified as showing long-term success (clinically relevant improvement on at least 4 out of 5 criteria) were compared with patients who were not classified as showing long-term success. Conceptually, the successful patients should have better values on each of the five single domains of Pain severity, Pain disability, Depressiveness, and Health-related quality of life (physical and mental) at the 12-month follow-up. This comparison is shown in Table 3, all five variables show a statistical difference of *p* < .001 analyzed with t-tests.

**Table 3:** Analysis of concurrent validity – Descriptive statistics at the 12-month follow-up

	Responder: improvement on 4 or more single criteria (change of at least ½ SD)	N	Mean	SD
Pain severity	No	102	57.93	(18.92)
	Yes	33	42.83	(17.76)
Disability through pain	No	102	3.39	(1.97)
	Yes	33	1.69	(1.44)
Depressiveness	No	102	7.07	(4.98)
	Yes	33	3.32	(2.70)
Physical HR-QoL	No	102	32.72	(8.46)
	Yes	33	39.40	(8.22)
Mental HR-QoL	No	102	36.57	(9.62)
	Yes	33	44.63	(8.01)

The mean change (therapy start to follow-up) in Pain severity was − 4.31 (SD 16.21) for non-responders and − 24.95 (SD 16.44) for responders (t (133) = 6.334; *p* < .001). The mean change in Pain disability was −.32 (SD 1.53) for non-responders and − 3.28 (SD 1.44) for responders (t (133) = 9.786; *p* < .001). The mean change in Depressiveness was −.70 (SD 4.51) for non-responders and − 6.44 (SD 4.22) for responders (t (133) = 6.462; *p* < .001). The mean change in Physical-health-related quality of life was + 3.39 (SD 7.32) for non-responders and + 11.61 (SD 7.62) for responders (t (133) = − 5.546; *p* < .001); for Mental-health-related quality of life, it was −.12 (SD 8.92) for non-responders and + 11.47 (SD 9.60) for responders (t (133) = − 6.368; *p* < .001).

In conclusion, 12 months after therapy, patients classified as successful showed significantly lower values on Pain severity, were significantly less disabled through their chronic pain condition, were significantly less depressive, and had significantly higher Mental- and Physical-health-related quality of life (all *p* < .001). Thus, each of the five single criteria alone was sensitive enough to reflect the differences between patients who were classified as successful versus not successful in the long term.

#### Criterion validity

**Fig. 2:**
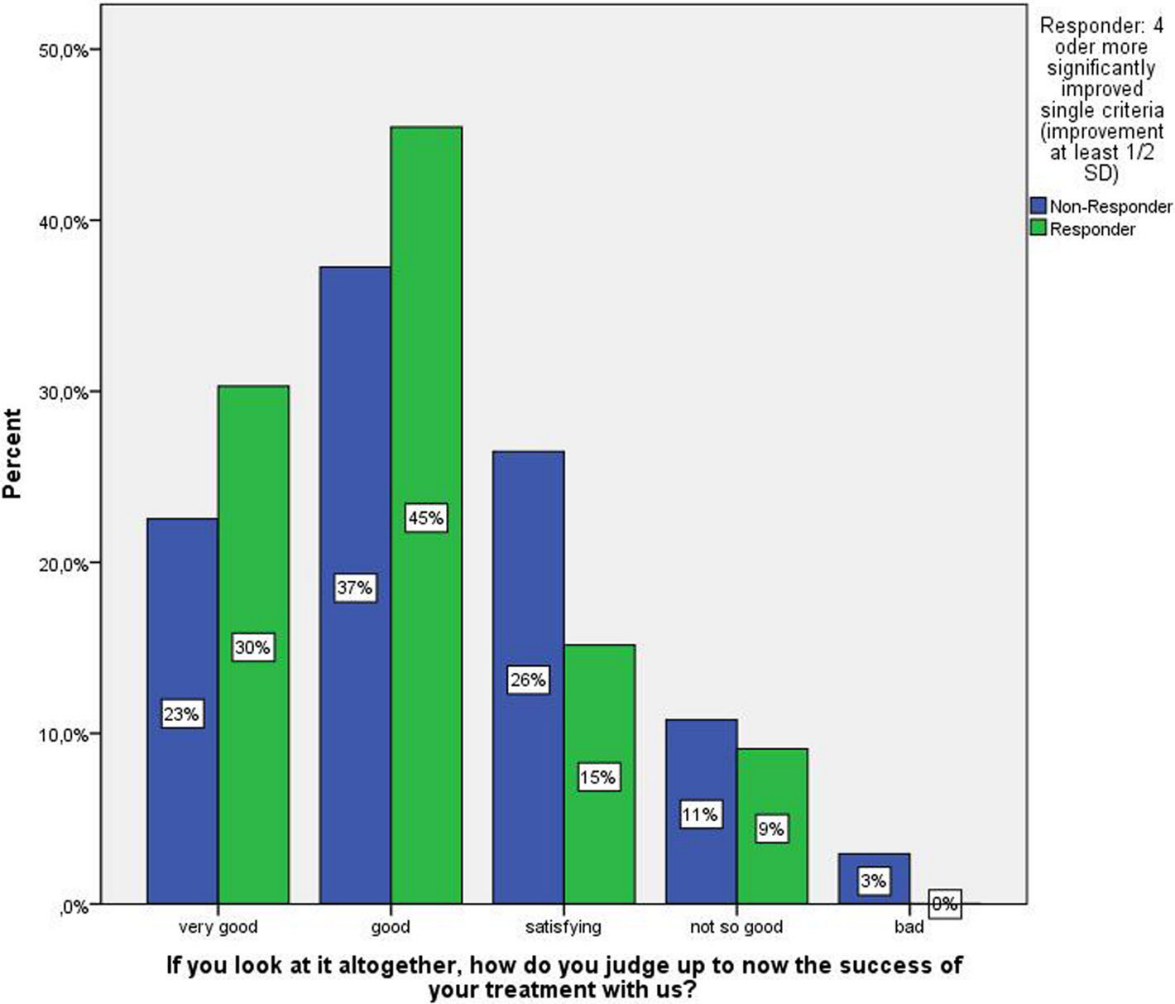
Differences in self-rated therapy success between patients classified as successful and not successful

For one measure of criterion validity, we conducted a comparison of responders/non-responders concerning the item “If you look at it altogether, how do you judge the success of your treatment with us up to now?” The self-rated success should show a clear association with the classification of being a responder or not via the combined success criterion. This was the case as Fig. 2 shows: While 75% of the patients classified as successful also self-rated their Therapy success as “very good” or “good” 12 months after treatment, the frequency for patients classified as non-responders was lower (60%). The mean of the Likert-scaled item (1 to 5) was tested for significant differences between the patients classified as “successful” (M = 2.03, SD = .92) and “non-successful” (M = 2.34, SD = 1.04). The difference did not reach statistical significance (t (133) = 1.545; *p* = .125).

We also used a second measure of criterion validity: Disability days at the 12-month follow-up. To reflect validity, the patients classified as successful would have significantly fewer Disability days (in the last 3 months) than patients classified as non-responders.

The t-test showed that the mean number of Disability days (15.31, SD = 23.15) in the group of responders was significantly lower than the mean number of Disability days in the group of non-responders (26.75, SD = 29.15); (t (133) = 2.308; *p* = .024) 1 year after therapy.

#### Convergent validity

We examined convergent validity with the help of three constructs: Stress, Anxiety, and Well-being. These constructs are not inherently the same as Pain variables, Depressiveness, or Quality of life (see also the “instruments” section) but are related to the single criteria that make up the combined success criterion (Pearson’s r correlation coefficients: see Table 4).

**Table 4:** Bivariate correlations of variables examined for convergent validity with the five domains of the combined success criterion (*N* = 135)

		Anxiety	Stress	Well-being
Pain severity	r	.442^**^	.511^**^	−.526^**^
Disability through pain	r	.416^**^	.536^**^	−.638^**^
Depressiveness	r	.569^**^	.675^**^	−.635^**^
Physical HR-QoL	r	−.366^**^	−.406^**^	.553^**^
Mental HR-QoL	r	−.535^**^	−.718^**^	.541^**^

^**^Statistically significant correlations (significance level *p* ≤ .01)

Thus, we compared the two groups of patients who were classified as successful or not successful over time on the above mentioned three constructs. While differences or change had to be expected in both groups due to therapeutic benefits – the patients classified as showing long-term success were expected to show more sustainable effects if the classification criterion was able to provide valid results.

##### Stress

**Fig. 3:**
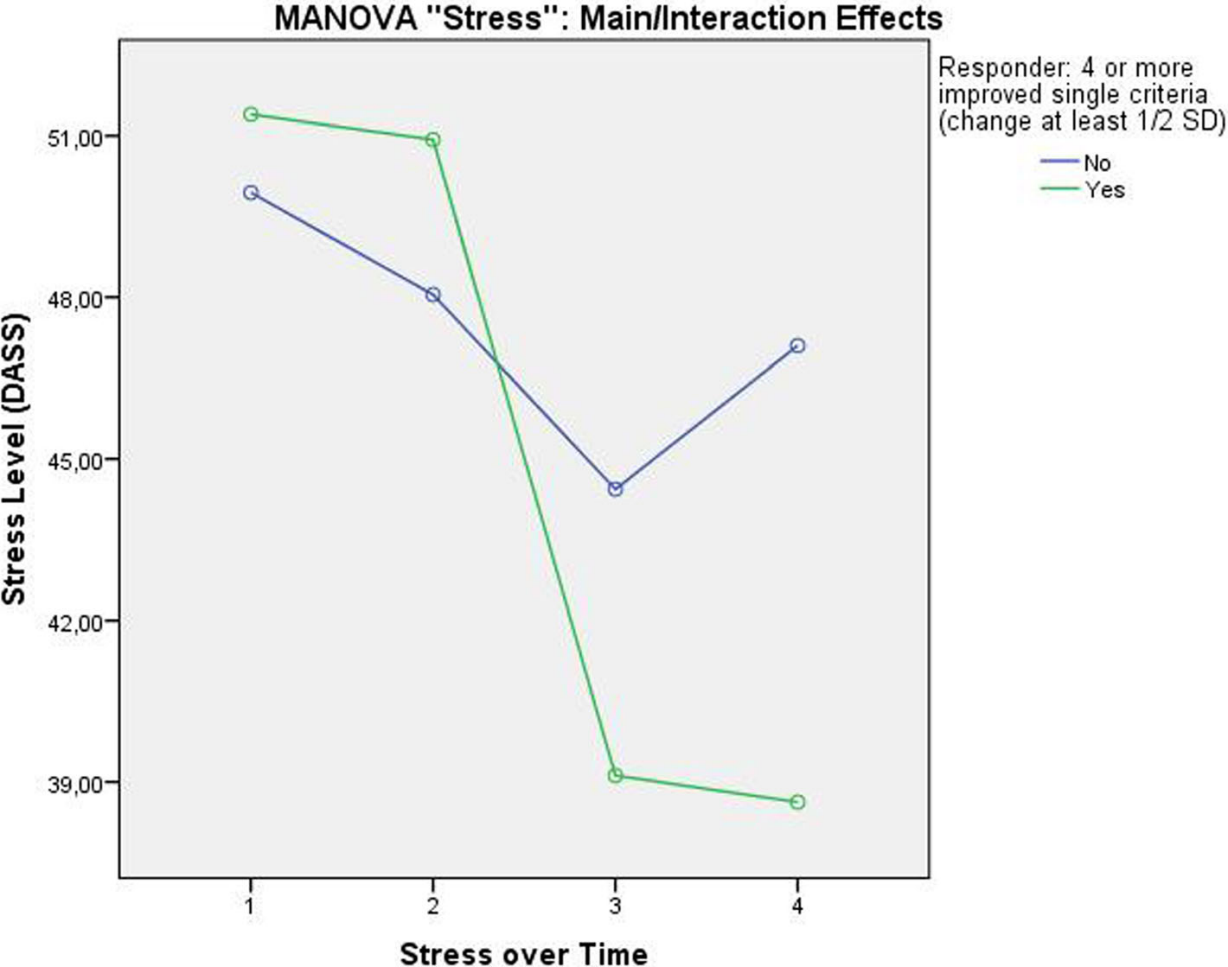
Stress Level over time differentiated by patients classified as successful and not successful

A MANOVA with the target variable Stress, the within-subject factor “Time” (4 time points: screening, therapy start, therapy end, 12-month follow-up), and the between-subject variable “Responder” (yes/no) showed a significant effect of Time (Pillai-Spur: F (3, 131) = 29.625, *p* < .001) and a significant interaction between Responder and Stress level over Time (Pillai-Spur: F (3, 131) = 9.907, *p* < .001). A main effect of the variable Responder could not be shown (F (1, 133) = 2.004, *p* = .159) because, conceptually, a significant main effect diminishes or is not interpretable when a significant interaction effect exists. These results indicate that the stress level decreased in both groups during the time they were in therapy, but in the group of patients classified as not successful, the stress level increased again after the therapy ended. The main and interaction effects are depicted graphically in Fig. 3.

##### Anxiety

**Fig. 4:**
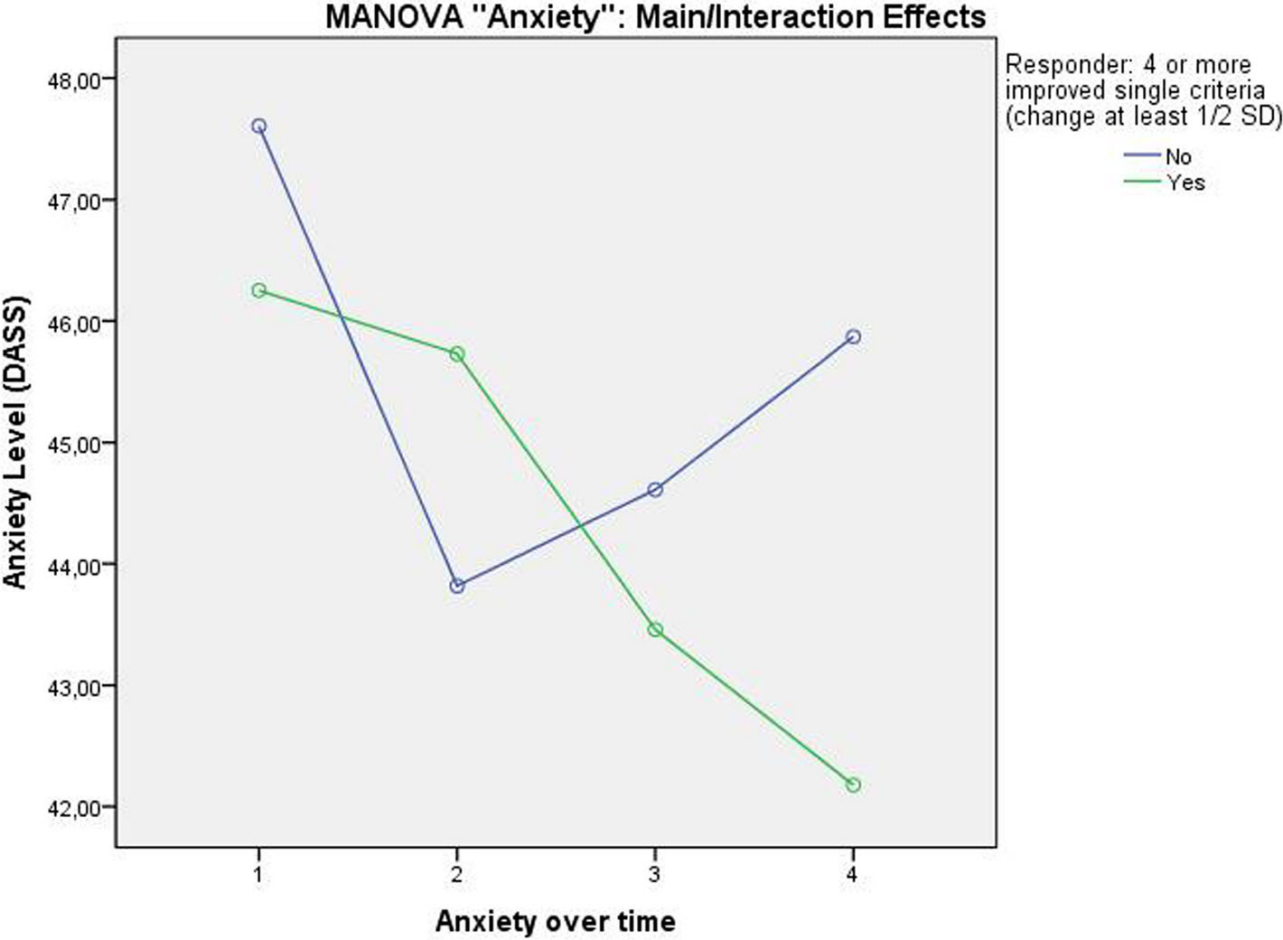
Anxiety Level over time differentiated by patients classified as successful and not successful

A second MANOVA involving the target variable Anxiety, the within-subject factor “Time” (4 time points: see above), and the between-subject variable “Responder” (yes/no) also showed a significant effect of the inner subject factor Time (Pillai-Spur: F (3, 131) = 5.825, *p* = .001) and a significant interaction between Responder and Anxiety level over Time (Pillai-Spur: F (3, 131) = 2.972, *p* = .034). As expected from this interaction effect, the main effect of the Responder variable was not significant (F (1, 133) = .506, *p* = .478). While the anxiety level of the responders decreased on a clinically relevant level during therapy and continued to fall slightly in the 12 months after therapy, the anxiety level of the patients classified as not successful showed a rather different trajectory (Fig. 4).

##### Well-being

**Fig. 5:**
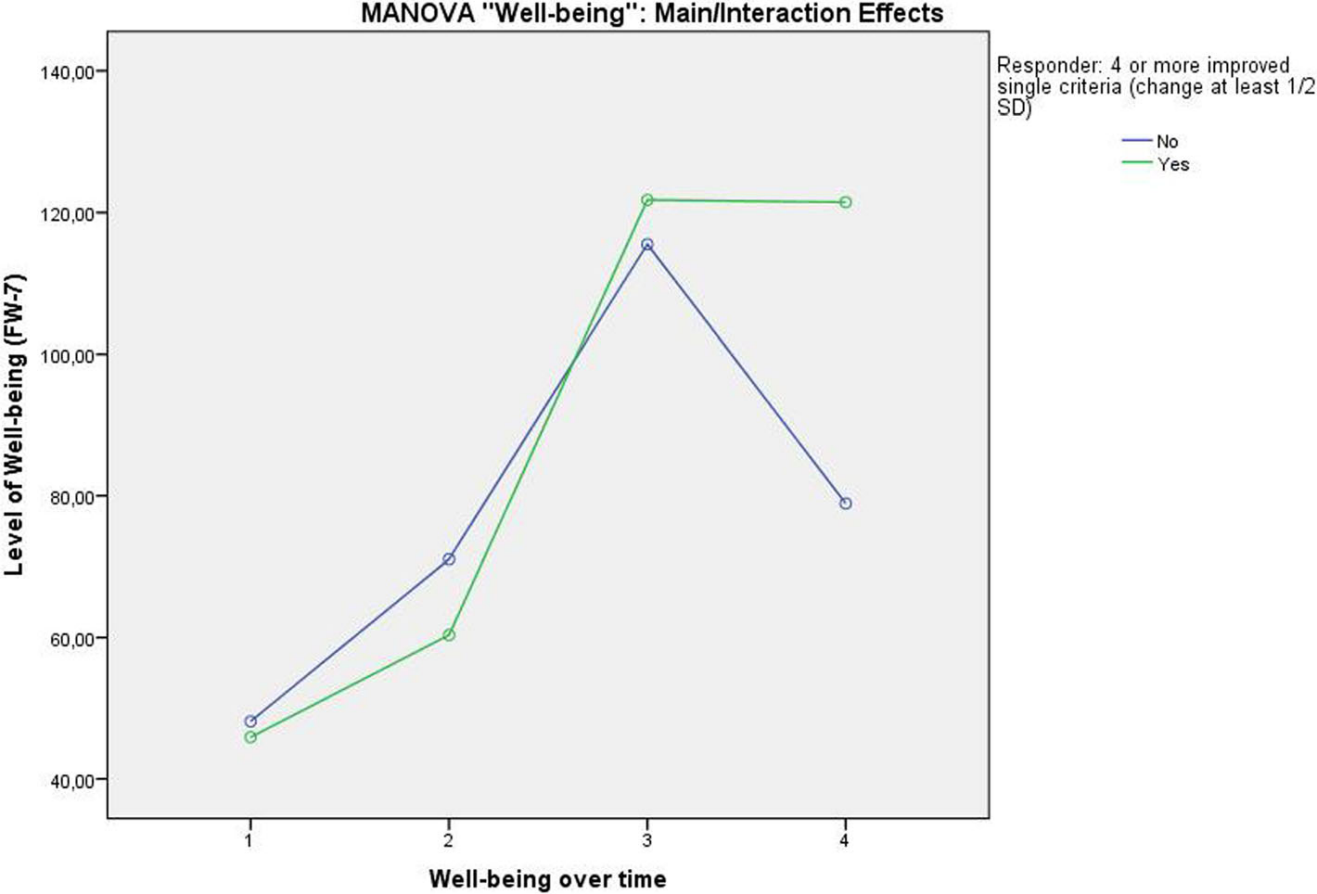
Level of Well-being over time differentiated by patients classified as successful and not successful

A third MANOVA was computed with the target variable Well-being. This resulted in a significant main effect of the within-subject factor Time (Pillai-Spur: F (3, 131) = 97.626, *p* < .001) and a significant interaction between Responder and the Well-being level over Time (Pillai-Spur: F (3, 131) = 9.594, *p* < .001). Again, due to the detection of an interaction effect, the main effect of the Responder variable was not interpretable or was nonsignificant (F (1, 133) = 1.830, *p* = .178). In the 12 months until the follow-up assessment, the level of well-being was stable for the patients classified as successful, whereas the other group showed a loss in well-being between the end of therapy and the 12-month follow-up (Fig. 5).

Thus, for the variables Stress, Anxiety and Well-being we were able to demonstrate convergent validity of the combined patient-reported-outcome criterion.

#### Discriminant validity

The combined success criterion for multimodal pain therapy based on patient-reported outcomes should conceptually not be related to the Chronicity stage of pain that characterizes patient collectives [6] and should be independent of co-morbid conditions [27]. Multimodal pain therapy is suitable for different chronicity stages, and virtually all patients with a chronic pain condition have psychic co-morbidities [28].

The Spearman correlation coefficient between the Chronicity stage according to Gerbershagen and the responder variable (0/1) was .094 (*p* = .280) and non-significant. Alternatively, the contingency coefficient was .114 (*p* = .618), which is also non-significant. The Gerbershagen Chronicity sum score, a second measure of chronicity, was also very weakly correlated with the combined success criterion (Eta = .110).

The Spearman correlation coefficient between the number of psychic comorbidities and the responder variable was .009 (*p* = .913). Alternatively, the Eta coefficient was .017. This is also a very weak/zero correlation.

Both analyses underscore the discriminant validity of the core patient-reported outcome measure set incorporated into the combined success criterion.

#### Summarizing the validation results

**Fig. 6:**
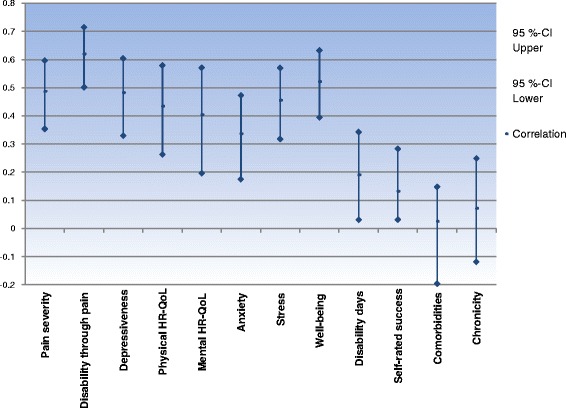
Absolute values of correlations between the combined success criterion and the different variables used to assess validity. The figure shows Spearman’s rho correlation coefficients and their confidence intervals generated by bootstrapping. For the following variables, a negative correlation with success was found: pain severity, disability through pain, depressiveness, anxiety, stress, disability days, self-rated success (due to measurement scaling), and comorbidities

To conclude our assessment of validity, all variables and scales covered in sections 1–4 of the results are depicted in Fig. 6, which shows their correlations with the combined success criterion (coefficients including confidence intervals). As expected, the success criterion’s highest correlations could be found with the variables that reflect concurrent validity (Pain severity, Disability through pain, Depressiveness) and convergent validity (Well-Being). Moderate correlations were shown by the remaining scales used to analyze convergent validity (Anxiety and Stress) but also some of the single variables of the combined success criterion (Health-related quality of life). The associations of the success criterion with variables operationalizing criterion validity (Self-rated Success, Disability days) were in the lower range. As reported, constructs presenting discriminant validity showed near-zero correlations (Comorbidities, Chronicity of pain).

#### Reliability

As a measure of reliability, we assessed the internal consistency of each of the five single criteria that make up the combined success criterion over the four time points: Cronbach’s alpha was .857 for pain severity, .905 for disability through pain, .899 for depressiveness, .870 for physical-, and .881 for mental-health-related quality of life.

It was not reasonable to determine retest reliability because of the questionable stability of successful treatment between the two assessment points: end of therapy and follow-up.

## Discussion

### Validation of the success criterion

The aim of the study - to validate a combined patient-reported outcome for success in multimodal pain therapy with long-term data - was realized with satisfactory results on different validity measures. To evaluate our validity estimates, we compared our results to those of other validation studies: Luttenberger et al. suggested a range between .2 and .5 for convergent validity in their validation study [29]. The three constructs we analyzed for convergent validity in our study showed correlations between .34 (anxiety) and .52 (well-being) with the success criterion and thus fell within the suggested range. In an instrument validation study by Luttenberger et al., constructs that were supposed to show convergent validity were found to correlate .39 to .43 with the outcome [30]. For discriminant validity, the association should not exceed .2 [29]. In our study, the absolute values of the two constructs operationalizing discriminant validity were .03 and .07, thus demonstrating the independence of the constructs. Other authors have reported values of .11 or .12 as representing discriminant validity [30].

For concurrent validity, studies in the literature have reported a wide range of correlation results depending on the instruments that were used for comparison. For example, Leonard et al. [31] reported concurrent validity values as high as .91 as the correlation between two instruments measuring functional limitation through pain with the same assessment method. However, medium-high correlations depicting concurrent validity have also been reported: .53 to .64 for the correlation between performance tests and rating scales [30]. Hoving and colleagues [32] compared instruments that all measured self-perceived pain disability and found correlations of .56 and .57 between the different constructs, thus supporting concurrent validity. In our study, concurrent validity correlation values ranged from .40 to .62 and were in the moderate range of correlations and thus comparable to the literature or somewhat lower for the variables representing health-related quality of life.

It seems surprising that the correlation of the success criterion with disability days (used for criterion validity) was in the lower range, whereas the inferential statistics showed a very clear difference between the patients classified as successful and those classified as not. This could be due to the large variance in the sample depicted in the standard deviations. On the other hand, correlation values between disability constructs and pain outcome measures close to .2 have also been reported in the literature: Wong and colleagues [33] validated the Survey of Pain Attitudes in Chinese and reported correlations between .16 and .37 in five of seven pain attitude scales from the ChSOPA-14 (next to lower correlations) with pain disability. In another instrument validation study, five of the eight scales of the Chronic Pain Coping Inventory showed correlations between .22 and .28 with pain disability [34]. Another bias that could possibly result in a low correlation between disability days and the combined success criterion is that the disability days are solely self-rated and are not objective measures like the number of days absent documented by employers or health-insurers.

### Objectivity evaluation

The combined success criterion for multimodal pain therapy fulfilled the measures of objectivity for a) Implementation -, b) Evaluation - and c) Interpretation objectivity:

- The success criterion was comprised of five single criteria (pain severity, disability through pain, depressiveness, health-related quality of life – physical and mental), each of which was assessed with a widely used and validated scale. For each scale, the procedures guaranteed the objective assessment of the construct.
- Each of the five scales operationalizing the five single criteria has standardized instructions concerning the handling of the single items and the generation of sum scores/standardized values.
- The published algorithm [6] that converts the results of the 5 single criteria into the combined success criterion is precisely defined. Two measurement points are needed to identify the change score. With the use of descriptive values (means and standard deviations), the interpreter can determine the number of single criteria on which a patient has shown clinically relevant improvement (at least ½ standard deviation). We discussed in detail the reasons for the ½ standard deviation change [6]. A dichotomous decision of whether a patient can be identified as successful or not can be made by applying the rule of at least 4 out of 5 criteria with a minimum improvement of ½ standard deviation by every interpreter.

### Limitations

The measurement of constructs such as pain severity as implemented in the German Pain Questionnaire has weaknesses as shown by a study that was conducted from the patient perspective [35]. This demonstrates that the suitability of existing instruments for chronic pain patients has to be taken into account when the validation results of this study are evaluated.

The analysis of validity and the outcome measurement itself were based on routine data as implemented in the routine care of multimodal pain therapy patients at the treatment center of the University Clinic Erlangen. On the one hand, this was an advantage because data were available on all treated patients, but this was also a disadvantage due to the inflexibility of the variable set. The variables available for analysis were strictly bound to the implemented German Pain Questionnaire, and other potentially interesting constructs for testing validity were not available for analysis. For example, it would have been interesting to evaluate the success classification against a clinical rating of a patient’s success made by a treatment expert.

Data of the validation study are based on one center. Thus the population of the pain treatment center in Erlangen cannot be seen as representative for all pain patients in Germany, especially since the treatment structure varies between in-patient and semi-in-patient multi-modal pain therapy within the therapy offers of the German Pain Centers.

Only patients’ self-ratings were available to operationalize long-term success. For a closer look at the health-economic perspective, it would be desirable to validate the criterion against objective data such as sick days. The question that could be asked would be whether patients classified as successful have significantly fewer sick days as a facet of criterion validity. In Germany, such data are administered and protected by health insurance companies. Thus, it was not possible to include such data in the routine data assessment 1 year after treatment. Of course, self-administered questionnaires and thus ratings of patients’ health status underlie multiple influences (inter-personal and intra-personal) and have the potential to be affected by judgment biases.

This study was not an efficacy or effectiveness study of multimodal pain therapy. Without a controlled design, it would not be appropriate to discuss the positive effects of multimodal pain therapy. The manuscript has to be understood as focusing on the validation of a classification measure based on a core outcome set used in multimodal pain therapy - even though there are hints about which constructs might be affected by multimodal pain therapy. The results concerning the associations of success with certain constructs were completely in line with the efficacy/effectiveness literature, and this is thus a sign that the multimodal pain therapy itself works as expected.

### Strengths & Perspectives

Even though this study is not about efficacy, it is among the few studies that have even reported a precise number of patients who can be seen as showing successful treatment in the long run. By defining a classification measure that groups treated patients into responders and non-responders a definite number of how many patients are classified as clinically relevantly improved results. Because multimodal therapies are important and are accepted, it is just and equitable to name and discuss the frequency of success. The rate of one fourth of the treated patients showing a clinically relevant improvement for at least 1 year after a semi-inpatient treatment is comparable to rates that have been achieved in other in-patient rehabilitation treatments with chronic patients, for example, in addiction treatment where the long-term success rate is about 32% [36].

The strength of the study is that it delivered one measurement that included five different outcome domains for pain patients a) that allowed us to classify patients into those who were successfully treated or not and b) were based on the most frequently used outcome domains in multimodal pain therapy thus far as identified by a systematic review [1]. The measurement instruments used to measure the five outcome domains are well-known, widely used, validated, and established. They are routinely implemented in all German Pain Treatment Centers.

The algorithm is easy to use, considers clinically relevant changes, is based on a widely used, methodological agreed-upon amount of change, has demonstrated its predictive value before [6], and now shows satisfactory validity results. Its objectivity and reliability are acceptable.

The use of the algorithm can be seen in the foreground in the following three fields of action:

1. By being able to classify patients as successful in the long term, an analysis of predictors of long-term success is possible. It generates new knowledge that is still incomplete for multimodal pain therapy about who profits and what factors are protective/resources for long-term success.
2. As a result of this analysis, one could adapt the allocation of treatment processes especially in the light of undersupply. As Ruan et al. [2] reported, there is - not only in Germany - a gap between the need for treatment with multimodal pain therapy and the availability of therapy with multimodal concepts. This study helps pave the way to more chances to provide intensive and expensive treatment with priority being given to those who will be likely to profit the most.
3. A closer analysis of the three quarters of the patients classified as less successful or not successful is possible and should be undertaken. The goal would be to identify factors that hinder long-term success and consequently provide information about how to appropriately adapt therapy routines and manuals. Kopf and Gjoni [37] emphasized the need for research in this field concerning the composition of treatment and its effects. Thus, greater efficiency in the implemented multimodal therapies could be achieved.

## Conclusion

The study aimed to validate a core outcome measure set that is based on patient-reported-outcome measures (PROMs) for use in multimodal pain therapy. The results of the validation analysis allow judging this success criterion as valid in operationalizing clinically relevant improvements. Thus, it can be seen as a step in answering questions of assessment of efficacy and effectiveness of multi-modal pain therapy depicted in the widely heterogenic use of outcome instruments. Furthermore, future research can build on this research to develop a set of predictors of success in multi-modal pain therapy.

## Additional file

Additional file 1: Baseline characteristics of the sample – group-specific analysis for patients classified long-term successful and not sucessful. Description of data: table showing group-specific sample description (descriptive statistics) for *N* = 133. (DOCX 14 kb)
