# Supplementary material for: Validation of a core patient-reported-outcome measure set for operationalizing success in multimodal pain therapy: useful for depicting long-term success?
Source: BMC Health Serv Res. 2018 Feb 17;18:117. doi: 10.1186/s12913-018-2911-6 (PMC5816476; doi:10.1186/s12913-018-2911-6)
Supplement: Supplementary file 1 — Baseline characteristics of the sample – group-specific analysis for patients classified long-term successful and not sucessful. Description of data: table showing group-specific sample description (descriptive statistics) for N = 133. (DOCX 14 kb) [file 12913_2018_2911_MOESM1_ESM.docx]

Table S1: Baseline characteristics of the sample – group-specific analysis for patients classified long-term successful and not sucessful

| **Variables** | **Responder (N = 33)** | **Non-responder (N = 102)** |
| --- | --- | --- |
| Age | 47.33 (11.23) | 52.20 (10.79) |
| Sex | % Females: 63.6 | % Females: 63.7 |
| Nationality | % Germans: 81.8 | % Germans: 87.3 |
| Marital status | % Married: 61.3 | % Married: 67.7 |
| Living alone | 21.2% | 12.7% |
| Living with partner | 71.0% | 76.3% |
| Living with children | 42.4% | 37.3% |
| Educational status | % Secondary general school (9 years): 48.5  % Secondary modern school (10 years): 18.2  % University entrance diploma (12+ years): 15.1 | % Secondary general school (9 years): 37.3  % Secondary modern school (10 years): 31.4  % University entrance diploma (12+ years): 8.9 |
| Apprenticeship | % yes: 78.8 | % yes: 85.3 |
| Employed | % yes: 72.7 | % yes: 64.7 |
| Sick Leave | % yes: 24.2 | % yes: 14.7 |
| Retirement payments | % yes: 6.1 | % yes: 17.6 |
| Recognized disability | % yes: 36.4 | % yes: 41.2 |
| Claim for recognition of disability | % yes: 15.2 | % yes: 6.9 |
